# How social background and interest in science are linked to junior high school students’ perceptions of the ecological transition

**DOI:** 10.3389/fpsyg.2024.1360166

**Published:** 2024-04-12

**Authors:** Kévin Nadarajah, Alain Somat, Céline Baeyens, Pascal Pansu

**Affiliations:** ^1^Laboratoire de Recherche sur les Apprentissages en Contexte (LaRAC), Univ. Grenoble Alpes, Grenoble, France; ^2^Laboratoire de Psychologie: Cognition, Comportement, Communication (LP3C), Psychology, Rennes 2 University, Rennes, France; ^3^Laboratoire Inter-Universitaire de Psychologie, Personnalité, Cognition, Changement Social (LIP/PC2S), Univ. Grenoble Alpes, Univ. Savoie Mont Blanc, Grenoble, France

**Keywords:** climate change, ecological transition perceptions, social representation, interest in science, social background, junior high school

## Abstract

Junior high school students are tomorrow’s key protagonists in the ecological transition. They need enlightened education to face the uncertainty and challenges of climate change. The development of climate change education programs requires a clear understanding of how young people perceive the issue. This study deals with social representations. Its aim was to understand how social background and interest in science are linked to the way young people perceive the concept of the ecological transition. Four hundred twenty-nine junior high school students took part in this study. Data were collected and subjected to prototypical analysis and factorial correspondence analysis. Three main findings emerged from the analysis: (1) the participants had significant knowledge of the ecological transition, (2) their awareness of the social aspects of climate change was limited, and (3) their representations of the ecological transition were linked to their interest in science and their parents’ social background. To conclude, these results underline the importance of educating all social classes about effective solutions for the ecological transition. Our findings also highlight the need to consider existing representations and prior knowledge when designing educational programs on climate change issues.

## 1 Introduction

Climate change is defined “*as the shift in climate patterns mainly caused by greenhouse gas emissions”* (Fawzy et al., 2020, p. 2,070). There are multiple consequences for humans and our ecosystems: extreme weather events, species extinction, food shortages, population displacement, and increased health risks, etc. (e.g., Pachauri et al., 2014; Haines and Ebi, 2019). To tackle these consequences, governments have repeatedly pledged to reduce their greenhouse gas emissions (e.g., Kyoto Protocol; 21st Conference of the Parties), undertaking to keep temperature rises below 2°C. These commitments have led to the choice of an ecological transition which, although based on ecological, economic, regulatory and social issues (Geels, 2011), has mainly focused on scientific and technological solutions in favor of ecology in Europe (e.g., Kemp et al., 2007; Kreinin, 2020). This choice necessarily involves developing scientific knowledge in order to encourage individual commitment to climate change (e.g., Fouad and Smith, 1996; Ojala, 2021), especially as 2,100, the impacts of climate change will have profoundly altered the health of the planet (Whitmee et al., 2015). Children born today will suffer the consequences of climate change throughout their lives (Watts et al., 2019), so they need to be given the scientific and technological tools that will enable them to adapt to tomorrow’s world (Lopoukhine et al., 2014).

The young people of today will be the main actors of tomorrow’s ecological transition (e.g., Kagawa and Selby, 2012; Burke et al., 2018; O’Brien et al., 2018; Cianconi et al., 2020). A study of 10,000 young people aged 16 to 25 in 10 countries (i.e., Australia, Brazil, Finland, France, India, Nigeria, Philippines, Portugal, the United Kingdom, and the United States) found that they questioned the consequences of climate change and seemed particularly uncertain about the future (Hickman et al., 2021). To overcome the inaction of previous generations and tackle the problem of climate change (Lee, 2013), they will need to be well informed (United Nations Framework Convention on Climate Change, 2015). Karsgaard and Davidson (2023) suggest that school is the place for the “*development of youth knowledge, creativity, efficacy and collective action skills in the face of climate change*” (p. 4). Educating young people about the challenges of climate change can give them the skills to cope with the forthcoming disruption and the power to act (Schreiner et al., 2005; Cambers and Diamond, 2010). However, as pointed out by Rousell and Cutter-Mackenzie-Knowles (2020), climate change education is a field that is in its infancy. While it is clear that young people today have strong feelings and considerable knowledge of climate change (Lee et al., 2020), little is known about how they perceive the ecological transition. According to the National Research Council (2012), developing climate change education “*begins with a clear picture of how students currently understand the issue*” (2012, p. 11).

Social Representation Theory (SRT; Moscovici, 1961) can help us to analyze the social construction of perceptions. This theory explains how individuals and groups give meaning to an issue, a risk or a social object (Höijer, 2011). In other words, how people build naive theories about their social environment (Jodelet, 1984), in order to attribute meaning to their world. These naïve theories are constructed around opinions, attitudes, beliefs and information related to an object or situation (Rateau et al., 2011), and their representations are linked to people’s social affiliations (Doise, 1990; Wagner et al., 1999; Joffe, 2003; Rateau et al., 2012). As social agents (Beauvois, 1984; Dubois and Pansu, 2021), individuals have a relationship with objects according to their cultural values (Howarth, 2006). This in turn gives them their social anchorage (Palmonari and Emiliani, 2016). These socially constructed representations can be studied according to their internal structures, in particular using the structural approach to social representations (Abric, 1994, 2003). According to this approach, social representations are made up of peripheral elements (i.e., dependent on the social contexts in which the individual evolves) organized around central elements (i.e., stable elements resulting from the history and ideology of the collective). Central elements have three functions: (1) signifying (i.e., the meaning given collectively by the group); (2) organizing (i.e., by repercussion on all the contents of the representation) and (3) stabilizing (subject to a strong consensus). Peripheral elements are based on the core consensus elements and express the variability of individual experiences in different social contexts. SRT therefore provides an understanding of how communication conveying scientific knowledge is transformed into common sense (Moscovici and Hewstone, 1984). SRT is an interpretative framework which may help to understand the representations of young people (Parrott et al., 2023) especially when it comes to environmental issues (Buijs et al., 2012).

The objective of this study was to: (1) analyze the representations of junior high school students (14–15 years old) in France with regard to the ecological transition; (2) understand how social and psychological filters structure the way they perceive the ecological transition. As socioeconomic status is a predictor of educational achievement (von Stumm et al., 2020), it has been hypothesized that the structuring of representations may depends on social determinants: such as parents’ socio-professional categories. Since scientific knowledge plays a central role in the ecological transition (Kreinin, 2020), it is also hypothesized that the participants’ interest in science contributes to the structuring of representations (Fouad and Smith, 1996). Finally, this paper aims to provide a stronger understanding of how young people’s representations of the ecological transition are structured.

## 2 Methods

### 2.1 Participants and procedure

The study proposal was reviewed and approved by the “Research Ethics Committee, Grenoble Alpes” (CERGA) of the University of Grenoble Alpes – Ethical approval number: Grenoble CERGA-Avis-2023-09. The study was carried out in partnership with the territorial services of the French Ministry of Education and conducted between May 2023 and October 2023. Four Hundred Thirty-nine junior high school students from 11 schools in the Auvergne Rhone Alpes region took part in the study (M*_age_* = 14.1, SD*_age_* = 0.619, range = 12–16, 53% female). Students were divided into three categories based on the socio-professional categories (SPC) of the first parent mentioned in the questionnaire. If the parents belonged to two different categories, participants were placed in the category corresponding to the higher SPC: SPC+ (self-employed trades professions; engineers; teachers; managers – 52.6%); SPC- (agricultural, factory and office workers – 37.8%); Inactive (students; retirees; unemployed – 6.4%). The three categories were based on information from the INSEE, which is the French Office for National Statistics and Economic Studies (see Supplementary materials for further details).

A few weeks before the study was carried out, the parents were asked to read an information leaflet and fill in the consent form to authorize (or not) their children’s participation. The researcher then visited the schools volunteering to carry out the study. The study took place in a computer room at predefined time slots. After presenting the study, the first author asked each pupil to go to a computer to complete an online ecological transition questionnaire on the Limesurvey^©^ platform.

### 2.2 Measures

#### 2.2.1 Perceptions of ecological transition

A free association task was used to collect perceptions of the ecological transition (e.g., Lo Monaco et al., 2016; Moliner and Lo Monaco, 2017). In this task, the junior high school students were presented with the stimulus words: “ecological transition.” On the basis of this induction, participants were free to associate four words or phrases that came to mind. This methodology yielded spontaneous associations from the participants.

#### 2.2.2 Interest in science

Fourteen items were used to measure interest in science adapted from Fouad and Smith (1996). This scale was originally developed for secondary school students to assess their interest in mathematics and science. Here, the mathematics items have been removed. Students were asked how much they liked to do certain things (e.g., Visit a science museum), rated from 0 (“I do not like it at all”) to 10 (“I like it very much”). The scores were aggregated. Internal consistency was satisfactory (α = 0.93).

### 2.3 Results

The corpus was composed of productions from 439 participants. A total of 1,578 verbal associations were collected from all participants, some of whom did not provide the expected 4 words. The corpus was cleaned up by the use of Excel^®^ (version 16.74) and Python^®^ (version 3.11), and then categorized by the authors, independently, using standard content analysis rules (Jones and Rosenberg, 1974; Dl Giacomo, 1980). 288 different words were obtained (167 are hapax, 57.98% of this corpus). Data were analyzed using JAMOVI^®^ (version 2.3.18), R^®^ (version 4.1.3), FactoshinySR (version 1.1 – Brosset and Delouvée, 2022) and two analyses were carried out.

Firstly, a prototypical analysis (Lo Monaco et al., 2016; Moliner and Lo Monaco, 2017; Delouvée et al., 2021), traditionally used in the structural approach to social representations, was conducted on the words given by the participants. This analysis was used to highlight the salience of elements in the representation by producing a table cross-referencing word frequency (i.e., below and above 10% of the number of evocations excluding hapaxes) and the order in which the word was produced, i.e., average occurrence rank (i.e., based on 2.5, the median of the four numbers of ranks – see Table 1).

**Table 1:** Prototypical analysis: results in terms of frequency and average importance associated with the categories of words reported by the participants for the stimulus words “ecological transition.”

		Average ranking					
		≤2.5			>2.5		
			*n*	*M*		*n*	*M*
*Frequency*	≥10%	Ecology	180	1,6	Nature	56	2.6
		Pollution	89	2.5	Economy	31	2.9
		Planet	64	2.4	Water management	19	2.9
		Change	61	2	Alternative transport	18	2.7
		Recycle	58	2.3	Future	15	2.9
		Waste sorting	52	2.3	Dams	13	2.6
		Wind turbine	39	2.2	Good for the planet	12	2.6
		Global warming	34	2.4	Preserving the environment	12	3
		Flora	36	2.3			
		Transition	28	2.1			
	<10%	Electric car	11	2.3	Fossil fuel	12	2.8
		Transformation	8	2	Nuclear	10	2.8
		Wasting	8	1.9	Climate	10	3.3
		Evolution	8	2.3	Better world	8	3.3
		Agriculture	8	2.5	Plastic	7	2.9
		Solar energy	6	2.5	Transfer	6	2.7
		Consuming less and better	6	2.5	Green Energy	5	2.6
		School	5	2.2	Cultivate	5	2.6
		Important	5	2.2	Develop	5	3
		Tidal turbines	5	2.2	Endangered species	5	3.4

“*n*” represents the total number of occurrences of the evocation and “*M*” represents the average appearance rank of the evocation.

Cross-referencing the evocations with the highest frequency and rank of appearance revealed three sub-categories (i.e., top left cell). The first category describes the causes and consequences of climate change (i.e., pollution, global warming). The second category describes the idea of moving toward a more ecological model (i.e., ecology, change, transition). Finally, the third sub-category expresses some of the best-known responses to the problem of climate change that have already been widely implemented (i.e., recycling, waste sorting, wind turbines). Moreover, for these young people, the main challenge of the ecological transition is to respond to climate change issues for the “planet” and its “flora.” These descriptive elements constitute the core representations of the ecological transition (e.g., Abric, 2003; Galli and Fasanelli, 2020). This suggests that young people are aware of the issues at stake in the ecological transition, both in terms of the problems it seeks to solve and the ways of achieving it.

The top right cell (peripheral; Abric, 2003) contains the frequent words, which do not appear quickly in the associative chain. Elements in this cell clarify the content evoked for the core. In fact, 2 categories can be identified. The first one concerns elements which refer to the idea of preserving natural environments for the future (i.e., nature, future, good for the planet, preserving the environment). The second category mentions solutions that are harder to implement in response to the consequences of climate change (i.e., water management, alternative transport, dams). Finally, it seems that for these young people, the “economy” is fundamental to the ecological transition.

The bottom left cell represents the contrasting elements that may not reach a consensus, but appear quickly in the associative chain, and are therefore considered very important by certain minority groups (Pianelli et al., 2010). Also, some individuals will emphasize the idea of turning to more virtuous models (i.e., transformation, evolution, consuming less and better). Participants also mentioned alternative solutions (e.g., tidal turbines, electric cars or solar energy, etc.). Finally, for the words in the second periphery (i.e., bottom right cell), i.e., the least salient of the peripheral system of representation that could be described as contextual (e.g., Delouvée et al., 2021), participants mention energy sources that produce greenhouse gasses (fossil fuels), and nuclear power, and their associated uses (i.e., plastics). Finally, participants express the idea of a better world as a possible consequence of the ecological transition. Although these elements are infrequent and unimportant, they seem to emerge in the content of the representation and are linked to the social context in which these young people evolve.

Secondly, a correspondence factor analysis (CFA; Benzécri, 1976) was carried out on participants’ evocations. This descriptive analysis was conducted to study how the words given by young people are associated with the parents’ socio-professional categories and the participants’ interest in science (Mouret et al., 2013; Nadarajah et al., 2022). In accordance with the work of Piermattéo et al. (2014) and the recommendations of Deschamps (2003), evocations whose frequency was greater than or equal to 6 were selected (*n* = 42 categories, 89.50% of the corpus without hapax). The relationship of these evocations with two variables was studied: (1) Interest in science was divided into two categories (“weak” and “strong”), which were defined with a distribution by the median 5; and (2) parents’ socio-professional categories were separated into 3 categories in accordance with the SPC groupings used for economic analyses by the French Office for National Statistics: SPC +, SPC −, and Inactive.

**Figure 1:**
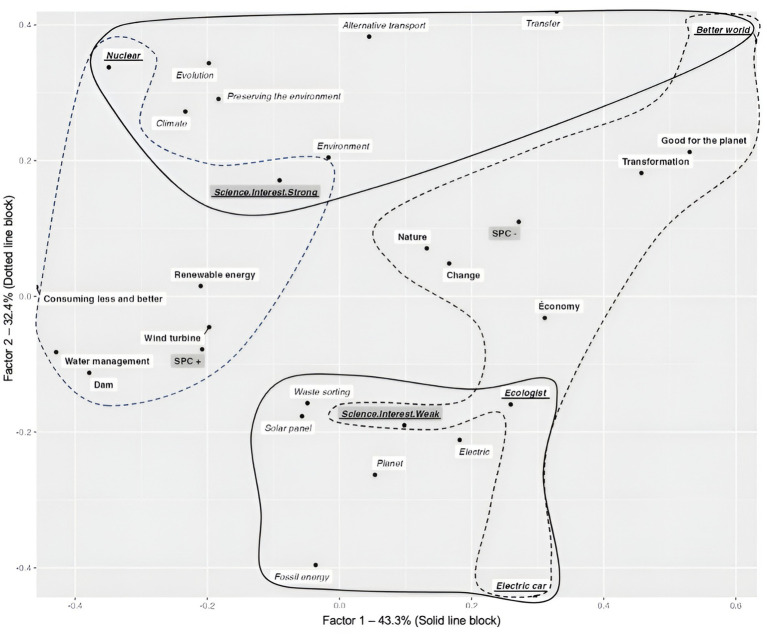
Graphical representation of the results produced by the factorial correspondence analysis for Factor 1 (Solid line block) and 2 (Dotted line block) concerning the “ecological transition” stimulus term. Shaded blocks refer to experimental conditions. The “Socio-Professional Category and Interest in Science variables” contribute to the formation of Factor 1; the “*Socio-Professional Category and Sciences Interest variables*” refer to the variables and measures that contribute to the formation of Factor 2; the “Socio-Professional Category and Interest in Science variables” refer to the variables and measures that contribute to the formation of Factors 1 and 2. “Perceptions” refers to perceptions that contribute to the formation of Factor 1; “*Perceptions*” refers to perceptions that contribute to the formation of Factor 2; “*Perceptions*” refers to perceptions that contribute to the formation of both Factors 1 and 2.

The CORR. F. A. highlights two factors that explain 75.70% of the table’s inertia (Factor 1 = 43.29%; Factor 2 = 32.41%). Factor 1 has a contribution from the terms of the variables “Socio-Professional Category”: *CF* (SPC+) = 0.07, *CF* (SPC−) = 0.10 “Science Interest”: *CF* (Science.Interest.Strong) = 0.37 + *CF* (Science.Interest.Weak) = 0.45, i.e., a contribution of 99%, to the formation of the factor. Factor 2 has a contribution from the terms of the variables “Socio-Professional Category”: *CF* (SPC+) = 0.36, *CF* (SPC-) = 0.47 “Science Interest”: *CF* (Science.Interest.Strong) = 0.07 + *CF* (Science.Interest.Weak) = 0.08, i.e., a contribution of 99%, to the formation of the factor. Figure 1 illustrates this configuration.

The vertical axis (factor 1) draws a distinction between young people according to their interest in science. Factor 1 indicates that the perceptions of participants with a strong interest in science differ from those of young people with a weak interest in science. Participants with a strong interest in science associate the ecological transition with the idea of evolution and the preservation of the environment. They also mention the changes in habits that are needed in order to achieve this (e.g., alternative transport). Finally, they mention “nuclear power,” probably because of its resurgence in the media as a “clean” energy that could enable the ecological transition. At the bottom of the vertical axis, the participants with a weak interest in science refer to individual actions to talk about the ecological transition (e.g., sorting waste). They are aware of the problem of climate change (e.g., planet) through the issue of fossil fuel scarcity. They also mention the “solar panel” as a technology that could play a part in the energy mix. However, they remain rather vague and descriptive when it comes to characterizing types of energy (e.g., electricity). Finally, they mention “ecologists” as being a category of the population that does not include themselves.

The horizontal axis (factor 2) shows young people according to their parents’ socio-professional category. Factor 2 indicates that the perceptions of participants whose parents belong to a high socio-professional category differ from those of young people whose parents belong to a low socio-professional category. Thus, participants whose parents belong to a high socio-professional category associate the ecological transition and the resolution of climate change with strategies or technologies to be implemented (i.e., water management, wind turbines, dams), or specific energy sources (i.e., renewable energy). Finally, these participants also refer to the idea of cutting through the old system of over-consumption to move toward more environmentally-friendly consumption (i.e., consuming less and better). On the other side of the axis, participants whose parents are in lower socio-professional categories use more general terms to refer to the ecological transition (i.e., change, nature, transformation, good for the planet). Furthermore, the “economy” takes on a predominant character for these participants when they think of the ecological transition.

## 3 Discussion

The aim of this study was to clarify the way the junior high school students perceive the ecological transition and to analyze their representations. Three mains’ results were obtained: the participants (1) have significant knowledge of the ecological transition and its underlying principles, (2) have little awareness of the social aspects linked to climate change, and (3) their representations of the ecological transition are structured by their interest in science and the socio-professional categories to which their parents belong.

Young people perceive the causes and consequences of climate change and link them to the need to switch to a more ecological model, through individual behaviors and technological solutions in favor of ecology (Kemp et al., 2007; Kreinin, 2020). Some research has shown that young people have misconceptions about climate change that persist despite the emphasis placed on climate change education (Jeffries et al., 2001). In this study, no misconceptions were observed. On the contrary, their representations of the ecological transition provide the foundations upon which learning in the classroom can be built. These results support the findings of Corner et al. (2015) showing that today’s 18-25-year-olds are probably the most well-informed age group. Nevertheless, in the corpus of this study, the social aspects inherent to climate change are barely mentioned. Indeed, the representations of the participants are oriented toward causes and consequences and have a technocentric vision of the ecological transition. For example, factors such as social justice, inclusion, citizen participation do not feature in the participants’ responses (e.g., Favreau, 2017; Huntjens, 2021). Another explanation for this technocentric vision could come from the chosen inductor term. The term “ecological transition” immediately implies thinking about the methods and technologies that could make this transition possible. However, other terms such as “sustainable development,” anchored in the three pillars: economic, environmental and social, could have enabled students to produce words related to social aspects (Mensah, 2019). Nevertheless, these factors should be considered when designing education programs for the ecological transition. As such, the concept of socio-ecological transition could provide an interesting theoretical foundation, while opening up a research program to study how it is perceived in non-scientific contexts (Larocque, 2023). Additionally, our results show that differences in the representational content are mainly linked to interest in science and the parents’ socio-professional category. Indeed, participants with a strong interest in science tend to describe the ecological transition by placing it in the broader context of environment preservation, while those whose interest in science is weaker only evoke solutions. Similarly, young people whose parents are in the low socio-professional category contrast with those whose parents are in the high socio-professional category. The former mentions a change for a better world, but remains relatively vague, whereas the latter develops strategic orientations and suggests technologies to break with an old system rooted in over-consumption. In both groups, the principles of objectification (transforming an abstract object to make it concrete), and anchoring (integrating the object into a pre-existing thought system), do not seem to have operated identically (Doise, 1990; Moliner, 2015). It is as if the objectifying and anchoring processes rely on psychological and social affiliations to elaborate the content of their representations of the ecological transition.

These results must be interpreted with the limitations in mind. First, the fact that there was only one time point of measurement calls for caution. Second, only 11 schools agreed to take part (out of 389). As a result, there may have been a sampling bias due to the school selection. To reduce this bias, we made sure that the schools were located in both urban and rural areas. Future studies could endeavor to recruit a greater number of schools in a wider geographical area.

To conclude, the ecological transition is a major issue for junior high school students, even if it manifests itself differently depending on their interest in science and the social group to which their parents belong. These results are important because new knowledge is embedded in a pre-existing system of representations. They can help us build a new way of designing educational programs by taking account of young people’s representations and prior knowledge of climate change issues. Today, young people do not need to be convinced of the reality of climate change, but it is essential to educate all social classes toward the development of a common knowledge base that will foster the implementation of solutions for the ecological transition. The ultimate goal is to educate all young people so that they are able to debate climate change issues in an organized and structured way, which would mean that they can (1) listen to each other’s differing viewpoints, and (2) work together constructively to develop strategies for the ecological transition. The principles of cooperative learning could be applied in order to facilitate productive ecological transition debates, thereby improving the knowledge acquisition process.

## Data availability statement

The raw data supporting the conclusions of this article will be made available by the authors, without undue reservation.

## Ethics statement

The studies involving humans were approved by Research Ethics Committee, Grenoble Alpes. The studies were conducted in accordance with the local legislation and institutional requirements. Written informed consent for participation in this study was provided by the participants’ legal guardians/next of kin. Written informed consent was obtained from the minor(s)’ legal guardian/next of kin for the publication of any potentially identifiable images or data included in this article.

## Author contributions

KN: Conceptualization, Data curation, Formal analysis, Methodology, Writing – original draft, Writing – review & editing. AS: Conceptualization, Methodology, Writing – review & editing. CB: Conceptualization, Methodology, Supervision, Writing – review & editing. PP: Conceptualization, Methodology, Supervision, Writing – review & editing.
