# Supplementary material for: How social background and interest in science are linked to junior high school students’ perceptions of the ecological transition
Source: Front Psychol. 2024 Apr 12;15:1360166. doi: 10.3389/fpsyg.2024.1360166 (PMC11046707; doi:10.3389/fpsyg.2024.1360166)
Supplement: Supplementary file 1 [file Table_1.DOCX]

Supplementary Material

## Supplementary material

*Science Interest items (adapted from Fouad & Smith, 1996)*

1. Work as an astronomer (studying stars and planets).
2. Take science courses.
3. Visit a science museum.
4. Listen to a speech by a famous scientist.
5. Create new technology.
6. Visit a science laboratory.
7. Sign up to a science association.
8. Read about scientific discoveries.
9. Take part in a science exhibition.
10. Work in a science lab.
11. Be at the origin of an invention in science.
12. Watch a science program on TV.
13. Acquire new knowledge in the fields of energy and electricity.
14. Work with chemistry equipment.

## Supplementary Tables

**Table 1.** Characteristics of the participants, means and (standard deviations) for each variable.

| **Variables** | **Overall (n=439)** | | |
| --- | --- | --- | --- |
| **Age** in year | 14.1 (.619) | | |
| **Gender** |  |  |  |
| Male | 199 | | |
| Female  Indeterminate | 235  5 | | |
| **Parents’ socio-professional category** |  |  |  |
| Self-employed trades professions, engineers, teachers, managers | 231 | | |
| Agricultural, factory and office workers | 166 | | |
| Students, retirees, unemployed | 28 | | |
| Missing data | 14 | | |
